# Associations between Feeling and Judging the Emotions of Happiness and Fear: Findings from a Large-Scale Field Experiment

**DOI:** 10.1371/journal.pone.0010640

**Published:** 2010-05-14

**Authors:** Tony W. Buchanan, David Bibas, Ralph Adolphs

**Affiliations:** 1 Department of Psychology, Saint Louis University, Saint Louis, Missouri, United States of America; 2 California Science Center, Los Angeles, California, United States of America; 3 Department of Biology and Department of Humanities and Social Sciences, California Institute of Technology, Pasadena, California, United States of America; Kyushu University, Japan

## Abstract

**Background:**

How do we recognize emotions from other people? One possibility is that our own emotional experiences guide us in the online recognition of emotion in others. A distinct but related possibility is that emotion experience helps us to learn how to recognize emotions in childhood.

**Methodology/Principal Findings:**

We explored these ideas in a large sample of people (N = 4,608) ranging from 5 to over 50 years old. Participants were asked to rate the intensity of emotional experience in their own lives, as well as to perform a task of facial emotion recognition. Those who reported more intense experience of fear and happiness were significantly more accurate (closer to prototypical) in recognizing facial expressions of fear and happiness, respectively, and intense experience of fear was associated also with more accurate recognition of surprised and happy facial expressions. The associations held across all age groups.

**Conclusions:**

These results suggest that the intensity of one's own emotional experience of fear and happiness correlates with the ability to recognize these emotions in others, and demonstrate such an association as early as age 5.

## Introduction

Recognition of facial affect is critically important in guiding social interactions, and humans are remarkably adept at recognizing a wide range of emotional cues from the faces of others. In spite of this general ability to recognize specific emotional cues, there is considerable individual variability in the recognition of facial affect. A number of clinical disorders, including focal brain lesions, autism [1], antisocial personality disorder [2], and mood disorders [3], are associated with altered facial affect recognition, and current research efforts are aimed at understanding why. Additionally, variables among the general population such as sex [4], general intelligence, age [5], and state variables such as mood [6] are known to influence facial recognition performance.

Despite the importance of the topic, both from a basic research and a clinical perspective, and despite the large number of ongoing studies, the mechanisms behind emotion recognition remain unclear. There is, however, no shortage of theories, all of which are debated. One possibility is that one's own ability to experience emotions is used in the recognition of another's facial expression, either through actual simulation of another's state [7] or through formulating a more cognitive theory about how another person feels and how they may behave [8]. A related possibility is that emotion experience, or knowledge of it, are not necessarily on-line for emotion recognition, but that emotion experience throughout development contributes to the ability to recognize emotions in others. Evidence for this experience-based learning of facial affect recognition comes from developmental psychology and neuropsychology. Children who have been abused show facilitated recognition of anger expressions, perhaps due to increased experience with negative emotion [9]. Paired deficits in both the experience and recognition of emotion for fear following amygdala damage [10], [11] and disgust following damage to the basal ganglia and/or the insula [12], [13] suggest that the neural substrates of emotion experience and recognition overlap, at least to some extent.

Despite these multiple sources of evidence for an association between the experience and recognition of emotion, it has been difficult to find direct support for the relationship in the normal population in general. If our own emotional experience influences how we recognize emotion in others, then there should be a reliable relationship between self-reported emotional experience, on the one hand, and the recognition of emotion in others, on the other hand. For instance, those individuals more sensitive to their own emotional states might also be more attuned to the emotions of others. However, given the many other, unrelated factors that contribute to one's own emotional experience in real life, the effect may be small and require large samples to detect it.

We carried out a large-scale initial study to probe these issues further. We collected data from a large sample of participants (N = 4,608), and across several different age ranges. We examined the possibility that those individuals who report having experienced strong emotional experiences in real life will also be most accurate in recognizing emotion in others. Here we define emotion recognition accuracy as closely matching a facial expression of emotion to a specified prototypical emotion expression, lying along a continuum of expressions. We show that those people who have experienced intense happiness are more accurate specifically in recognizing facial expressions of happiness in others, and that those who have experienced intense fear are more accurate in recognizing facial expressions of fear, as well as to some extent recognizing other emotions.

## Results

**Figure 1:**
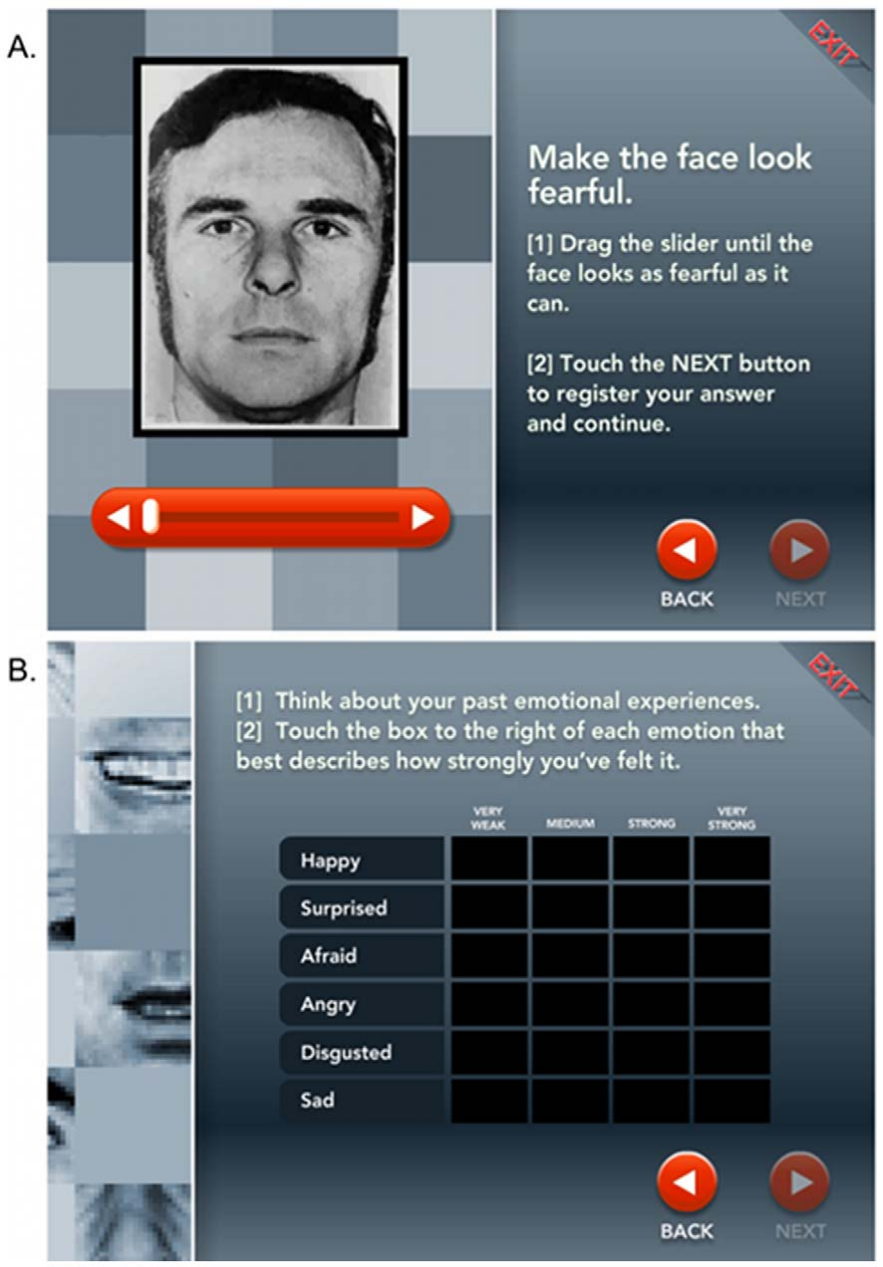
Stimuli Used in the Study. Example screenshots of A) the facial affect recognition task and B) the emotional experience task.

We tested 4,608 participants spanning ages 5 through over 50 in the context of a traveling exhibit installation of the California Science Museum (Table 1). Two pieces of data were collected from each participant: (1) their self-rated experience of emotion in everyday life, and (2) their accuracy in judging the emotion of morphed facial expressions, from moving a slider to dynamically change the face image to correspond to a stated emotion label (see Figure 1). Participants were divided into 4 groups on the basis of their emotion experience: Very Weak, Medium, Strong, and Very Strong. Inspection of the raw data distributions of slider placement during the emotion recognition task by each of these four emotional experience groups showed that every group had unimodal distributions, with the modal response for every emotion being the ‘accurate’ emotion prototype as defined by the experimenter (with the exception of disgust; see comment in Materials and Methods below). However, those groups with weaker emotion experience had distributions that became progressively more flat in both directions, with a substantially higher proportion of responses further from the prototype (see Figures S1 and S2 in Supporting Information).

**Table 1:** Frequency counts of female and male participants within each age group.

Age Groups	Female	Male	Totals
Age 5–10	548	420	968
Age 11–16	961	604	1565
Age 17–20	268	130	398
Age 21–30	330	193	523
Age 31–40	296	185	481
Age 41–50	255	198	453
Over 50	118	102	220
Totals	2776	1832	4608

**Figure 2:**
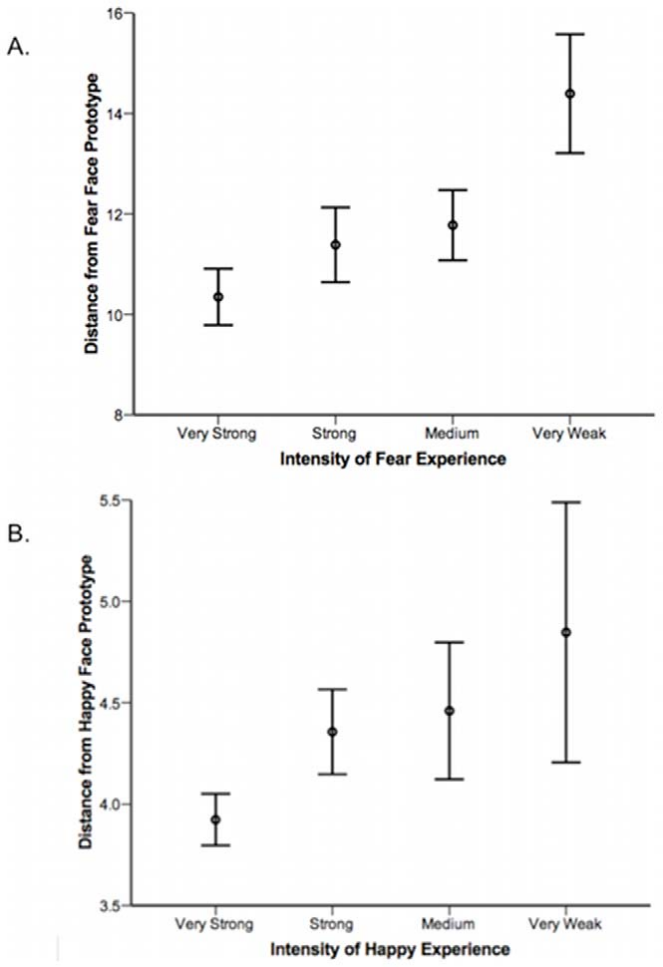
Emotional Experience is Associated with Facial Affect Recognition. Association between the intensity of emotion experience (x-axis) and recognition of facial affect (y-axis). Data show the mean and 95% confidence intervals for the absolute value of the distance from the prototypical expression for each experience group. A: fear; B: happiness.

Given the possibility of age and sex differences, we included these factors in our analyses (see Table 1 for age group breakdown and number of participants of each sex in each group). For each emotion category, a 2 (Sex) ×6 (Age Group: ages 5–10, 11–16, 17–20, 21–30, 31–40, 41–50, Over 50)×4 (Emotion Experience; Very Weak, Medium, Strong, Very Strong) ANOVA was conducted, with the absolute value of the distance from each prototypical emotion as the dependent variable as a measure of accuracy. We found a significant effect for fear and happiness: participants who reported experiencing ‘very strong’ fear or happiness were more likely to show accurate facial recognition of fear and happiness, respectively, than those who reported ‘very weak’ fear experiences (Fear: F(3,4552) = 7.7, p<0.0001, eta squared  = 0.005; Happy: F(3,4552) = 4.5, p<0.01, eta squared  = 0.003; see Figure 2). Post-hoc comparisons showed that people who reported experiencing very weak fear rated fear faces significantly less accurately than all the other emotion experience groups (ps<0.0001, Bonferroni corrected). Furthermore, those who reported experiencing very strong happiness rated happy faces significantly more accurately than all the other emotion experience groups (ps<0.05, Bonferroni corrected). Anger experience showed a trend toward predicting anger recognition (Anger: F(1,4552) = 2.3, p = 0.08, eta squared  = 0.002). Follow up contrasts did not show significant differences among the anger recognition groups, however (ps>0.15). Experience of surprise was not significantly predictive of surprise recognition performance (Surprise: F(1,4552) = 1.5, p = 0.2, eta squared <0.0001).

**Figure 3:**
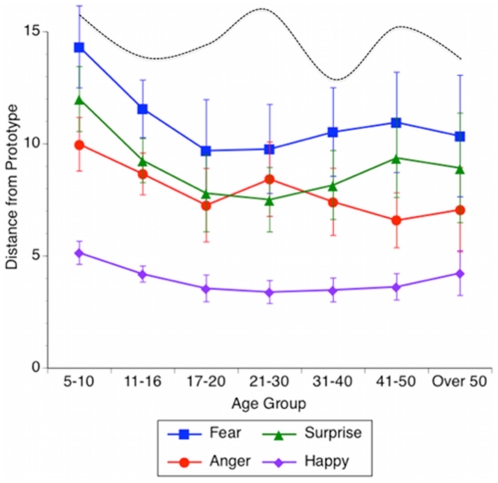
Emotional Experience Effects are Consistent Across Age Groups. Absolute value of the distance from the prototypical expression for all emotions across age groups. Data show mean and 95% confidence intervals. Dashed line indicates the distance from the prototypical expression of fear from those who report having experienced ‘Very Weak’ fear in their lives.

There was a significant effect of age across all emotion recognition categories, (F(6,4552)>5.0, ps<0.0001, eta squared >0.007; see Figure 3). Follow-up contrasts showed that this effect was primarily due to the youngest age group (ages 5–10) showing the least accurate facial affect recognition (ps<0.05 compared to all other age groups, Bonferroni corrected; see Figure 3). Participants in the ‘Very Weak’ experience groups across all age ranges showed the poorest recognition performance of all emotion recognition categories. There was, however, a significant Age × Emotion Experience interaction for fear recognition (F(18,2552) = 2.0, p<0.01, eta squared  = 0.008) but none of the other emotion recognition categories. This interaction may be due to the especially poor and highly variable fear recognition of those reporting ‘Very Weak’ fear experience across all age groups. The performance of this group was highly variable, ranging from an average of 11.1 to 18.2 morphs away from the fear prototype across the various age groups (see dashed line in Figure 3). These effects were not due to a preponderance of the youngest participants in the ‘Weak Fear Experience Group’, as these participants were distributed throughout the fear experience groups (see Table S1 in the Supporting Information). There were no significant Age by Emotion Experience interactions found for recognition of any of the other emotion categories, further suggesting that the influence of emotional experience on facial affect recognition holds across all age groups. There were no significant effects of sex on affect recognition performance, nor were there significant interactions between sex and emotional experience. Females were more likely than males to report ‘very strong’ experiences of all emotions tested: happiness (64% vs. 56%), fear (38% vs. 28%), surprise (28% vs. 26%), and anger (48% vs. 46%). Because the effects of emotional experience on facial affect recognition were independent of sex, we have chosen to omit further discussion of sex differences.

Given the strong effect of fear experience on fear recognition, we also examined associations between the experience of fear and recognition of the other facial emotions (happy, surprise, and anger). People who reported stronger experience of fear showed more accurate recognition of surprise (F(3,4552) = 4.8, p<0.002, eta squared  = 0.003) and happiness (F(3,4552) = 2.7, p<0.05, eta squared  = 0.002). Post-hoc comparisons showed that people who reported experiencing very weak fear rated surprised and happy faces significantly less accurately than those who reported experiencing very strong fear (ps<0.05, Bonferroni corrected). Fear experience was not a significant predictor of anger facial expression accuracy (F(3,4552)<1, p>0.7, eta squared <0.0001).

## Discussion

Our study demonstrates for the first time that in the general population emotional experience in real life is reliably associated with the ability to recognize happiness and fear in others. Very weak experiences of both these emotions were associated with less accurate recognition of those particular emotions from the face. Fear experience was further associated with more accurate recognition of happiness and surprise. These findings support the hypothesis that own emotional experience may play a role in recognizing the emotions of other people, either through on-line simulation or through effects during development.

There are several possible explanations for the effect we found. Participants may have implicitly called on their own experience with a particular emotion in order to choose which facial configuration best matched their understanding of how a particular emotion is expressed. Those individuals who reported having a ‘very strong’ experience of fear, for example, may have more ready access to their own facial configuration during a fear experience and therefore more closely match the visual stimulus of fear with the prototypical expression. Recent work has suggested that one's affective empathy may be a trait-like characteristic, which interacts with the expressivity of others to influence accuracy in labeling the affective expressions of others [14]. Another alternative is that the relationship between reported emotional experience and emotional recognition may reflect the affective beliefs of an individual, rather than the momentary experience of emotion. Retrospective assessments of emotional experience (as used in the current study) are thought to be an index of an individual's beliefs about their emotional states and may not necessarily reflect actual experiences [15], [16]. As such, it is possible that those individuals reporting high experience of fear may differ from their low-fear counterparts more in terms of their beliefs about emotions and less in terms of actual emotional experience. It is also possible that the reaction to others' expressions is influenced by tempermental characteristics present at birth. Temperament is known to influence the expression of emotion [17]. The emotional reaction to another's expression may be determined by a combination of temperamental influences on reactivity coupled with a more nuanced understanding of expressions that develop through learning.

Another possible explanation for these findings is that those who reported having ‘very weak’ emotional experiences may have a different conception of what a fearful or happy face may look like. In either case, stronger experience of emotion may influence an individual toward a more ‘modal’ or prototypical understanding of facial expressions of emotion, making the individual more likely to accurately interpret the social cues of others. Without this experience-enhanced recognition, an individual may not recognize signals from another either as quickly or as accurately.

This idea that we recognize other people's emotional expressions through simulation of the emotion experience has a history in philosophy and psychology with simulationist models of emotion recognition [7]. These models argue that accurately recognizing emotion from the faces of others may require the experience (either concurrently or through past experience) of that particular emotional state. Similarly, one common model of empathy suggests that observing another's emotional state activates representations of that emotion in the observer [18]. These representations then, may activate the bodily states associated with that emotion merely by observing another's expression. The establishment of representations of these emotional states should take place through experiential learning. Individuals often experience intense emotion in the presence of others. Experience in these social emotional settings may give observers experience with how other people's faces react in a frightening situation, for example. In the absence of such experiences, that individual may be less able to accurately recognize a given emotional expression through a reduced ability to represent the experience of that emotion. Results from our study are consistent with the hypothesis that those individuals who have not had a strong experience of either fear or happiness may be less able to represent those particular emotions, and when required to match their own representation of these emotions to facial expressions of these stimuli, are less able to match them to prototypical expressions. A final and simpler possibility is that those people who are more sensitive to their own emotions would report more intense emotional experiences, and would also be more accurate in their recognition of emotion in others. At this stage, our finding clearly demonstrates an association, but the causal relationship between experience and recognition of emotions remains to be investigated in detail.

The specificity of the association between emotional experience and recognition for only fear and happiness deserves comment. The experience of happiness was associated only with the recognition of happiness, and the experience of fear was associated primarily with recognition of fear, although also (at least marginally) with the recognition of surprise and happiness. Such specificity would support versions of simulation theory in which the simulated emotion is more precisely matched to the observed emotion. The strong experience of fear may also lead to a facilitation of the processing of ambiguous emotional signals from others [19].

A final important issue is the development of facial affect recognition throughout childhood and adolescence. Since we included participants spread from age 5 through over 50 (Table 1), our study was in a particularly good position to examine whether there were any notable changes across these different ages. An individual's history of emotional experience may play a role in determining his or her response to, and appreciation of, facial affect. Extreme examples of this association come from psychiatric disorders such as antisocial personality disorder [2] and depression [3], which are both associated with impaired recognition of facial affect, as well as from developmental disorders such as autism. But pathological experiential effects do not always result in impaired performance: children who have been physically abused are faster at categorizing prototypical facial expressions of anger than are typically developing children [9]. In addition to these influences of mental disorders and physical abuse, other factors such as mood within the normal range may influence the speed and accuracy in recognition of an emotional expression within a healthy population [6]. In our study, we found evidence for reduced affect recognition accuracy among the youngest participants (aged 5–10) for all emotions, but importantly the pattern of association between experience and recognition was the same across age groups. Importantly, the stimuli for participants of all ages were the same adult face series, which may have influenced performance among the younger groups of participants. Future work should address this issue by examining facial affect recognition in a wide age-range of participants and using facial stimuli from models that vary in age. These findings indicate that although there is a general age-related improvement in affect recognition, this pattern stabilizes by middle childhood and the effects of emotional experience are observed at the earliest age range tested.

### Conclusion

In a large sample of museum-goers, we examined the relationship between emotional experience and emotional recognition. We demonstrate that people who reported experiencing strong fear or happiness show more accurate (closer to prototypical) recognition of expressions of these emotions, respectively. Further, fearful experience was correlated with more accurate facial affect recognition across the emotions of happiness and surprise. Our results may provide a basis for individual differences in emotion recognition, empathy, and other aspects of social behavior.

## Materials and Methods

### Ethics

The procedures described in this manuscript conform to the guidelines of the Institutional Review Board of Saint Louis University. Informed consent was not obtained from participants because the data were collected and analyzed anonymously.

### Participants

Participants were visitors to *Goosebumps!: The Science of Fear*, a traveling exhibit at the California Science Center in Los Angeles, California, The Center of Science and Industry (COSI) in Columbus, Ohio, and The Liberty Science Center in Jersey City, New Jersey between June of 2007 and July of 2008. The exhibit, developed by the California Science Center, was a hands-on demonstration focusing on the biology, psychology, and sociology of emotion, with an emphasis on fear. Data presented in the current study were drawn from a computerized component designed by R. Adolphs as part of the larger exhibit in which visitors could choose freely to participate.

A total of 4992 participants fully completed the exhibits described in this study. From this total, 384 participants (7%) were excluded because their scores on at least one of the facial affect recognition scales were >3 standard deviations from the mean; this resulted in a final total of 4608 participants included in all analyses (see Table 1 for demographic information). Participants were asked to provide their sex, race, and age (choices were ages 5–10, 11–16, 17–20, 21–30, 31–40, 41–50, and over 50); no identifying information was collected. The sample was ethnically diverse, including Caucasians (62%), Latino/Hispanics (21%), Asian Americans (11%), and African Americans (6%). Inclusion of information about race and age are included to demonstrate the diversity of the sample included in this analysis. As we had no *a priori* hypotheses about these variables on facial affect recognition or emotional experience, we do not present analyses examining these variables.

### Emotion Recognition Task

Figure 1A shows a screenshot of the Emotion Recognition Task. Face stimuli were displayed on a monitor with the instructions “Make the face look angry [or one of the other emotions]. Drag the slider until the face looks as angry [or one of the other emotions] as it can.” A slider scale located under the face allowed the participant to scroll through continuous morphs between expressions in order to change the expression on the face to match a given label. The stimuli consisted of 255 frames showing prototypes and morphs of expressions from the Ekman and Friesen series [20]. Participants were allowed to explore the full range of facial expressions by sliding the slider bar through a fixed sequence of neutral, happy, surprised, fearful, anger, disgust, and sadness from left to the right side of the slider scale. Participants failed to recognize disgust well, perhaps because the prototypical expression of disgust does not map well onto the lay idea of this concept [21]. There were a total of 36 intermediate morphing steps between 2 frames of each prototypical facial expression. Each participant was asked to match the emotions to labels using the slider in a different, random order for the emotions happy, surprised, fear, anger, and disgust. So, for example, when a subject was asked to “make the face look happy”, he/she was required to move the slider scale until he or she decided that the face matched his or her conception of what a happy face should look like. Contrary to the typical emotion morphing tasks, which include a separate morph series for each emotion ranging from neutral at one end and the target emotion on the other end, our task included all emotions along the same continuum.

We chose the order of our morphing continuum based on prior studies showing that expressions are ranked in this order in terms of their perceived similarity [11], [22]. We used this task for two reasons: first, it is more interesting for the subject to perform than the standard task of matching a given facial expression to an emotion label or rating it; second, we felt that the fine-grained nature of the emotion morphs together with the interactive aspect of scrolling through them would yield a more accurate and unbiased match between the emotion label and expression.

#### Scoring

Performances on this task were scored as the absolute value of the difference of each participant's slider placement from the prototypical facial expression (given in the number of morph steps from the prototype corresponding to the label to be matched).

### Emotional Experience

Figure 1B shows a screenshot from the Emotional Experience task. After completing the facial affect recognition task, participants were given the following instructions: “Think about your past emotional experiences. Touch the box to the right of each emotion that best describes how strongly you've felt it.” The emotions were fear, happy, surprise, and angry. The boxes were labeled: Very Weak, Medium, Strong, and Very Strong. We examined the association between the emotions assessed in both the recognition task and the experience questions: fear, happiness, surprise, and anger.

## Supporting Information

Figure S1 Fear Experience Shapes Facial Fear Recognition. Histograms depicting the distributions of distance from fear face prototype separated by reported fear experience. X-axes represent distance from fear face prototype (prototype located at 0 on x-axis). Y-axes represent the number of participants from each group who chose a particular face morph when asked to “make the face look fearful.”
(0.17 MB TIF)

Figure S2 Distributions of Facial Affect Recognition. Histograms depicting the distributions of the raw slider placements for A: happy, B: surprise, C: fear, and D: anger. X-axes represent the numerical location of the slider placement relative to the prototype for each expression. Y-axes represent the number of participants across the whole sample who chose a particular face morph. Arrows on each x-axis denote the location of the prototypical facial expression for each emotion.
(0.48 MB TIF)

Table S1 Age Distribution Across Fear Experience Groups. The table shows the age distributions of the different fear experience groups (numbers indicate the number of participants within each age group who were in each fear experience group).
(0.03 MB DOC)

## References

[1] Adolphs R (2002). Recognizing emotion from facial expressions: psychological and neurological mechanisms.. Behavioral and Cognitive Neuroscience Reviews.

[2] Marsh AA, Blair RJ (2008). Deficits in facial affect recognition among antisocial populations: a meta-analysis.. Neurosci Biobehav Rev.

[3] Leppanen JM (2006). Emotional information processing in mood disorders: a review of behavioral and neuroimaging findings.. Curr Opin Psychiatry.

[4] Kring AM, Gordon AH (1998). Sex differences in emotion: expression, experience, and physiology.. J Pers Soc Psychol.

[5] Herba C, Phillips M (2004). Development of facial expression recognition from childhood to adolescence: behavioural and neurological perspectives.. J Child Psychol Psychiatry.

[6] Chepenik LG, Cornew LA, Farah MJ (2007). The influence of sad mood on cognition.. Emotion.

[7] Goldman AI, Sripada CS (2005). Simulationist models of face-based emotion recognition.. Cognition.

[8] Frith CD, Frith U (2006). The neural basis of mentalizing.. Neuron.

[9] Pollak SD, Sinha P (2002). Effects of early experience on children's recognition of facial displays of emotion.. Dev Psychol.

[10] Calder AJ, Young AW, Rowland D, Perrett DI, Hodges JR (1996). Facial emotion recognition after bilateral amygdala damage: differentially severe impairment of fear.. Cognitive Neuropsychology.

[11] Adolphs R, Tranel D, Damasio H, Damasio AR (1995). Fear and the human amygdala.. J Neurosci.

[12] Sprengelmeyer R, Young AW, Calder A, Karnat H, Lange V (1996). Loss of disgust. Perception of faces and emotions in Huntington's disease.. Brain.

[13] Calder AJ, Keane J, Manes F, Antoun N, Young AW (2000). Impaired recognition and experience of disgust following brain injury.. Nature Neuroscience.

[14] Zaki J, Bolger N, Ochsner K (2008). It takes two: the interpersonal nature of empathic accuracy.. Psychol Sci.

[15] Robinson MD, Clore GL (2002). Belief and feeling: evidence for an accessibility model of emotional self-report.. Psychol Bull.

[16] Barrett LF (1997). The relationship among momentary emotional experiences, personality descriptions, and retrospective ratings of emotion.. Pers Soc Psychol Bull.

[17] Izard CE, Abe JA (2004). Developmental changes in facial expressions of emotions in the strange situation during the second year of life.. Emotion.

[18] Preston SD, de Waal FB (2002). Empathy: Its ultimate and proximate bases.. Behav Brain Sci.

[19] Whalen PJ (1998). Fear, vigilance, and ambiguity: Initial neuroimaging studies of the human amygdala.. Current Directions in Psychological Science.

[20] Ekman P, Friesen W (1976). Pictures of Facial Affect..

[21] Nabi RL (2002). The theoretical versus the lay meaning of disgust: implications for emotion research.. Cognition and Emotion.

[22] Russell JA (1980). A circumplex model of affect.. Journal of Personality and Social Psychology.

