# Supplementary figures and images for: Associations between Feeling and Judging the Emotions of Happiness and Fear: Findings from a Large-Scale Field Experiment
Source: PLoS One. 2010 May 14;5(5):e10640. doi: 10.1371/journal.pone.0010640 (PMC2871050; doi:10.1371/journal.pone.0010640)

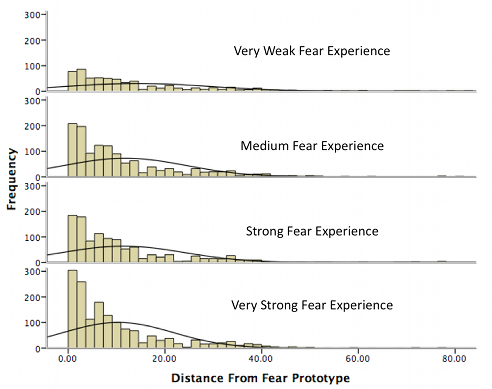

Supplement: Figure S1 — Fear Experience Shapes Facial Fear Recognition. Histograms depicting the distributions of distance from fear face prototype separated by reported fear experience. X-axes represent distance from fear face prototype (prototype located at 0 on x-axis). Y-axes represent the number of participants from each group who chose a particular face morph when asked to “make the face look fearful.” (0.17 MB TIF) [file pone.0010640.s001.tif]

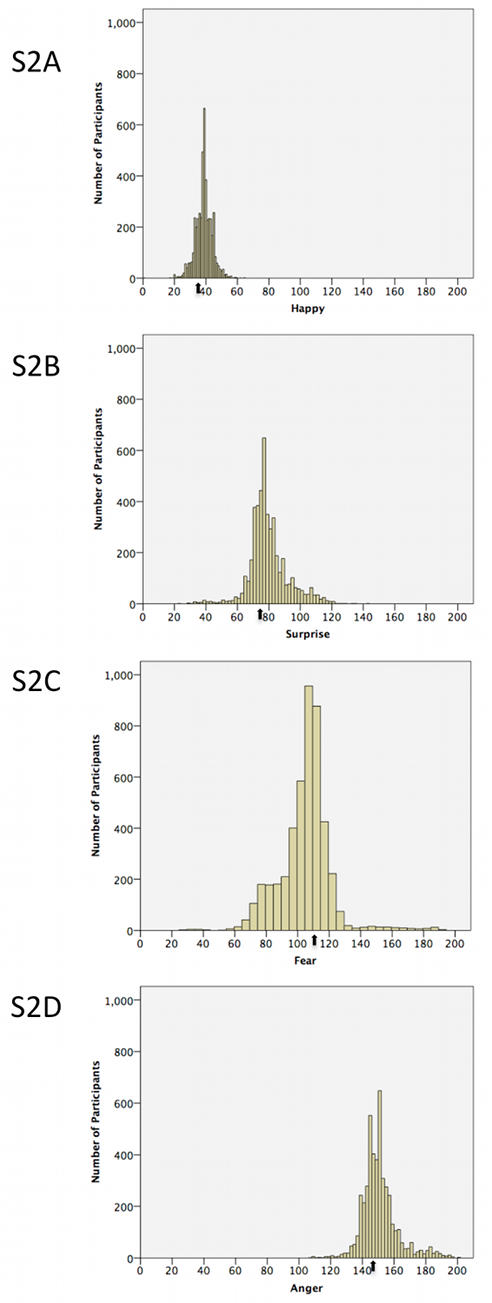

Supplement: Figure S2 — Distributions of Facial Affect Recognition. Histograms depicting the distributions of the raw slider placements for A: happy, B: surprise, C: fear, and D: anger. X-axes represent the numerical location of the slider placement relative to the prototype for each expression. Y-axes represent the number of participants across the whole sample who chose a particular face morph. Arrows on each x-axis denote the location of the prototypical facial expression for each emotion. (0.48 MB TIF) [file pone.0010640.s002.tif]
