# Supplementary material for: Associations between Feeling and Judging the Emotions of Happiness and Fear: Findings from a Large-Scale Field Experiment
Source: PLoS One. 2010 May 14;5(5):e10640. doi: 10.1371/journal.pone.0010640 (PMC2871050; doi:10.1371/journal.pone.0010640)
Supplement: Table S1 — Age Distribution Across Fear Experience Groups. The table shows the age distributions of the different fear experience groups (numbers indicate the number of participants within each age group who were in each fear experience group). (0.03 MB DOC) [file pone.0010640.s003.doc]

|  | **Fear Experience** | | | |
| --- | --- | --- | --- | --- |
| **Age Groups** | **Very Weak** | **Medium** | **Strong** | **Very Strong** |
| Age 5-10 | 199 | 268 | 171 | 330 |
| Age 11-16 | 239 | 445 | 398 | 483 |
| Age 17-20 | 48 | 115 | 100 | 135 |
| Age 21-30 | 51 | 135 | 140 | 197 |
| Age 31-40 | 39 | 145 | 132 | 165 |
| Age 41-50 | 42 | 113 | 119 | 179 |
| Over 50 | 18 | 57 | 58 | 87 |
| Totals | 639 | 1278 | 1118 | 1576 |
